# Mitochondrial abnormalities and low grade inflammation are present in the skeletal muscle of a minority of patients with amyotrophic lateral sclerosis; an observational myopathology study

**DOI:** 10.1186/s40478-014-0165-z

**Published:** 2014-12-14

**Authors:** Safa Al-Sarraj, Andrew King, Matt Cleveland, Pierre-François Pradat, Andrea Corse, Jeffrey D Rothstein, Peter Nigel Leigh, Bams Abila, Stewart Bates, Jens Wurthner, Vincent Meininger

**Affiliations:** Neuropathology, Neuroscience Academic Building, Kings College Hospital, Denmark Hill, London, SE5 9RS UK; Biopharm Translational Medicine, GSK, Stevenage, UK; Département des Maladies du Système Nerveux, APHP, Reseau SLA IDF Groupe Hospitalier Pitié-Salpêtrière, Sorbonne Universités, UPMC Univ Paris 06, INSERM, CNRS, Laboratoire d’Imagerie Biomédicale (LIB), Paris, F-75005 France; Department of Neurology, Johns Hopkins School of Medicine, 1800 Orleans Street, Baltimore, MD USA; Division of Medicine (Neurology), Brighton and Sussex Medical School, Trafford Centre for biomedical Research, Falmer, Brighton BN1 9RY UK; Novartis Oncology Translational Medicine, Basel, Switzerland

**Keywords:** Amyotrophic lateral Sclerosis, Mitochondria, Inflammation, Pathology and Muscle

## Abstract

**Background:**

Amyotrophic lateral sclerosis (ALS) is a primary progressive neurodegenerative disease characterised by neuronal loss of lower motor neurons (in the spinal cord and brainstem) and/or upper motor neurons (in the motor cortex) and subsequent denervation atrophy of skeletal muscle.

**Aim:**

A comprehensive examination of muscle pathology from a cohort of clinically confirmed ALS patients, including an investigation of inflammation, complement activation, and deposition of abnormal proteins in order to compare them with findings from an age-matched, control group.

**Material and methods:**

31 muscle biopsies from clinically confirmed ALS patients and 20 normal controls underwent a comprehensive protocol of histochemical and immunohistochemical stains, including HLA-ABC, C5b-9, p62, and TDP-43.

**Results:**

Neurogenic changes were confirmed in 30/31 ALS cases. In one case, no neurogenic changes could be detected. Muscle fibre necrosis was seen in 5/31 cases and chronic mononuclear inflammatory cell infiltration in 5/31 (2 of them overlapped with those showing muscle necrosis). In four biopsies there was an increase in the proportion of cytochrome oxidase (COX) negative fibres (2-3%). p62 faintly stained cytoplasmic bodies in eight cases and none were immunoreactive to TDP-43.

**Conclusion:**

This large series of muscle biopsies from patients with ALS demonstrates neurogenic atrophy is a nearly uniform finding and that mild mitochondrial abnormalities and low-grade inflammation can be seen and do not rule out the diagnosis of ALS. These findings could lend support to the notion that ALS is a complex and heterogeneous disorder.

## Introduction

Amyotrophic lateral sclerosis (ALS) is a progressive neurodegenerative disease characterised primarily by degeneration of upper and lower motor neurons (LMN) in the cerebral cortex, spinal cord and brainstem. This leads to relentlessly progressive weakness and widespread flaccid paralysis and/or spasticity, depending on the predominance of upper versus LMN disease [1,2].

The presentation is heterogeneous and may begin with appendicular weakness, bulbar weakness or respiratory muscle weakness. Neuro-imaging is useful in excluding other diseases. Electromyography (EMG) is used to support the clinical findings, possibly allowing a more accurate and early diagnosis, as it can detect denervation sometimes before clinical signs are evident. (El Escorial and Awaji criteria) [3].

Pathologically, ALS is characterised by neuronal loss in the anterior horn of the spinal cord, brainstem nuclei, and Betz cells of the motor cortex, and also by the deposition of abnormal ubiquitinated inclusions immunoreactive to TDP-43 [4]. But these central nervous system changes are only accessible post mortem. In contrast, the muscle pathology of denervation atrophy, such as angular, atrophic fibres, grouped atrophy and fibre type grouping can be assessed pre-mortem and may lead to earlier diagnostic certainty or provide a means to assess possible treatment impact on disease progression. It is therefore very important to understand muscle pathology in ALS for clinical trials in this field.

The aetiology and pathogenesis of the neuronal death in ALS remains poorly understood. The majority of ALS cases are sporadic while 10-15% are familial. Mutations have been identified in genes encoding for Cu/Zn superoxide dismutase (SOD1), VAMP (vesicle associated membrane protein), angiogenin (ANG), TDP-43 and Fused in sarcoma (FUS), Optineurin (OPTN), and more recently C9orf72 (linked to chromosome 9) [5-10].

There are several theories to explain the neurodegenerative processes including glutamate-induced excitotoxicity, axonal transport impairment, proteasome dysfunction, aberrant functioning of glial cells, alterations in muscle and neuromuscular junction [11,12], and mitochondrial dysfunction [13-18]. There are reports of abnormal aggregation of mitochondria and impaired respiratory chain function (particularly in complex 1) in experimental mice with SOD1 mutation [5,19-21]. However, the role of mitochondrial abnormalities in the pathogenesis of ALS is unknown [22]. Although some reports suggest mitochondrial dysfunction in the skeletal muscle and a significant increase in mitochondrial DNA deletions indicate a primary defect in muscles of ALS patients; others suggest that mitochondrial dysfunction is a consequence of the motor neuron cell death [8,11,23-28]. The majority of reports describing the muscle pathology of ALS patients are case reports; systematic investigations of muscle pathology in a large cohort of patient with ALS are few [29-33].

We report the pathological changes in the skeletal muscle in a cohort of 31 patients with ALS according to El Escorial criteria and compare them with muscle biopsies from 20 age-matched controls.

## Material and methods

### Study populations

A total of 32 patients with ALS (age range 27–75 years, mean age 54.8 years) were recruited as part of a methodology study (NOG111329) to assess biomarkers of ALS; 31 subjects provided muscle biopsy samples. ALS patients with a diagnosis of clinically definite or probable ALS (according to El Escorial diagnostic criteria, revised according to the Airlie House Conference 1998) were recruited at 3 ALS centres. Inclusion criteria included onset of muscle weakness within 24 months of study entry and MRC score in the deltoid muscle of MRC grade 3 or 4. Subjects with evidence of other neuromuscular disorders were excluded. Twenty healthy volunteers (age range 41–69 years, mean age 55.7 years) were recruited at the Guy’s Drug Research Unit (GDRU) in London to assess levels of potential ALS biomarkers in muscle samples from healthy subjects (NOG113240).

The study protocol, protocol amendments, informed consent and any other information that required pre-approval were reviewed and approved by a national, regional or investigational centre ethics committee or an institutional review board. This study was conducted in accordance with Good Clinical Practice and the guiding principles of the Declaration of Helsinki, and all subjects provided written informed consent.

### Muscle biopsy procedure

Thirty-one ALS patients and 20 healthy volunteers underwent open muscle biopsy from the deltoid muscle according to standard procedures. For ALS patients, the weaker of the two deltoid muscles was selected. A muscle specimen of an approximate size of 1.0 cm × 0.5 cm × 0.5 cm was taken per subject.

### Processing muscle biopsy samples

The muscle biopsies were processed freshly (i.e. unfixed) in the laboratory soon after the surgical removal to minimize preparative artefact. The muscle biopsy sample was divided perpendicular to the muscle fibres into two equally large pieces, one piece for pathological assessment, while the second piece was processed for other biomarkers (not reported here). The pathology sample was oriented and fixed transversely to cork discs with OCT and then snap frozen in iso-pentane cooled on dry ice. Tissue was transferred to pre-cooled cryotubes and stored frozen at -80°C.

### Histology and immunohistochemistry

Serial cryostat sections were cut from the frozen biopsy material (7 mm thickness) and stained for H&E, ATPase 9.4, ATPase 4.6, ATPase 4.2, nicotinamide adenine dinucleotide tetrazolium reductase (NADH-TR), succinic dehydrogenase (SDH), cytochrome oxidase (COX) with and without SDH, Gomori trichrome (GT), acid phosphatase (AP), Periodic acid-Schiff (PAS), Periodic acid-Schiff with diastase (PAS-D), myophosphorylase and Sudan Black.

Further immunohistochemistry stains were performed for p62 (BD transduction Labs, clone: 3/p62 LCK Ligand, dilution 1:150), pTDP-43 (Cosmo Bio Co. LTD, code TIP-PTD-P02, dilution1:1500), HLA-ABC (Dako, clone: W6/32, dilution 1:1200) and C5b-9 “membrane attack antigen” (Novocastra, clone: aE11, dilution 1:200). CD3(Dako, code M7254M dilution 1:50), CD20(Dako code M075501, dilution 1:200), CD8(Novocastra code CD8-295-CE, dilution 1:20), CD 4 (Novocastracode CD4-1f6-CE-S, dilution 1:20), neonatal myosin (Novocastra code NCL-MHCn, dilution 1:5) and Utrophin (Novocastra code NCL-DRP2, dilution 1:100).

Cryostat sections were processed according to the protocol at the Department of Clinical Neuropathology, King’s College Hospital London. Slides were interpreted by a senior Neuropathologist (SALS) blind to clinical information and findings are summarised in Tables 1 and 2.

**Table 1:** **Summary of muscle pathology in ALS/MND patients**

**Age/sex**	**NC**	**Infl**	**Nec**	**COX -ve**	**HLA**	**C5b-9**	**P62**	**TDP**	**CK**
54 M	+	+	-	-	-	+ F	+	-	350
						+ C			
54 M	+	-	+	-	+	+ F	+	-	107
75 F	+	-	+	2%	+	+ F	+	-	114
56 M	+	-	-	-	-	-	-	-	443
61 F	+	-	-	-	-	-	-	-	285
57 M	+	-	-	-	-	+ F	+	-	337
27 M	+	-	-	-	-	+ F	+	-	313
45 M	+	-	-	-	-	-	-	-	151
57 F	-	+	-	-	-	+ F	-	-	47
						+ C			
36 M	+	-	-	-	-	-	-	-	143
29 M	+	-	-	-	-	-	+	-	170
66 M	+	-	-	3%	-	-	-	-	82
69 M	+	-	-	-	-	+ F	-	-	267
66 M	+	-	-	-	+	+ F	-	-	119
61 M	+	-	-	-	-	+ F	-	-	807
60 M	+	-	-	-	-	+ F	-	-	295
59 F	+	+	+	-	-	-	-	-	145
34 M	+	+	+	-	+	+ F	-	-	1386
47 F	+	-	-	-	-	+ F	-	-	108
41 M	+	-	-	-	-	+ F	-	-	238
67 M	+	-	-	1%	-	+ F	-	-	148
40 M	+	-	-	-	+	+ C	+	-	166
60 F	+	-	-	3%	-	+ F	-	-	252
61 F	+	-	-	2%	-	+ F	-	-	99
55 M	+	-	-	-	-	+ F	-	-	391
50 M	+	+	-	-	-	+ F	-	-	963
67 F	+	-	-	-	-	+ F	+	-	52
61 F	+	-	-	-	-	+ F	-	-	318
65 M	+	-	-	-	-	+ F	-	-	150
48 F	+/−	-	+	-	-	-	-	-	130
67 M	+	-	-	-	-	-	-	-	425

NC: neurogenic changes; Infl: inflammation; Nec: Necrosis. C: capillaires. F: fibres.

**Table 2:** **Summary of muscle pathology in control patients**

**Age/sex**	**NC**	**Infl/nec**	**COX -ve fibre**	**HLA**	**C5b-9**	**P62**	**TDP**
56 F	-	-	-	-	-	-	-
54 M	-	-	-	-	-	-	-
42 M	-	-	-	-	-	-	-
53 F	+	-	-	-	-	-	-
67 M	-	-	-	-	-	-	-
69 M	-	-	<1%	-	-	-	-
67 M	-	-	<1%	-	-	-	-
41 M	-	-	-	-	-	-	-
49 M	-	-	-	-	-	-	-
41 M	-	-	-	-	-	-	-
43 F	-	-	-	-	-	-	-
65 M	-	-	-	-	-	-	-
65 M	-	-	-	-	-	-	-
57 M	-	-	-	-	-	-	-
50 M	-	-	-	-	-	-	-
51 M	-	-	-	-	-	-	-
51 F	-	-	-	-	-	-	-
64 M	-	-	< 1%	-	-	-	-
67 F	-	-	-	-	-	-	-
62 F	-	-	-	-	-	-	-

NC: neurogenic changes; Infl: inflammation; Nec: Necrosis. C: endomysial capillaires. F: fibres.

### Laboratory assessments

Plasma (EDTA) samples were collected from ALS subjects prior to collection of open muscle biopsy samples and aliquots dispensed and frozen at -80°C. Frozen samples were shipped to The Doctors Laboratory (TDL) and an exploratory analysis performed for lactate dehydrogenase (LDHI2), creatinine (CREJ2) and creatine kinase (CK) using the Roche Cobas system. Note that sample stability data was not available to cover the storage time (>1 year) or conditions (−80°C) for these samples.

Non parametric Mann Whitney test was carried out to find the correlation between increased CK levels and muscle fibre necrosis and inflammation.

## Results

Thirty of 31 ALS muscle biopsies showed evidence of neurogenic atrophy with various stages of denervation and re-innervation of muscle fibres. These included early changes such as scattered angular, atrophic fibres which are densely stained with NADH-TR as well as more chronic changes with small and large grouped atrophy and fibre type grouping. One case showed no clear features of neurogenic changes but definite ALS on clinical grounds. There were occasional small fibres and lymphocyte infiltration limited to the perimysium, however, there was no endomysial inflammation, the HLA-ABC was not over-expressed and creatinine kinase was low (CK: 47 IU) suggesting that the focus of inflammatory cells was a non-specific finding and that inflammatory myopathy was unlikely.

### Neurogenic changes

**Figure 1:**
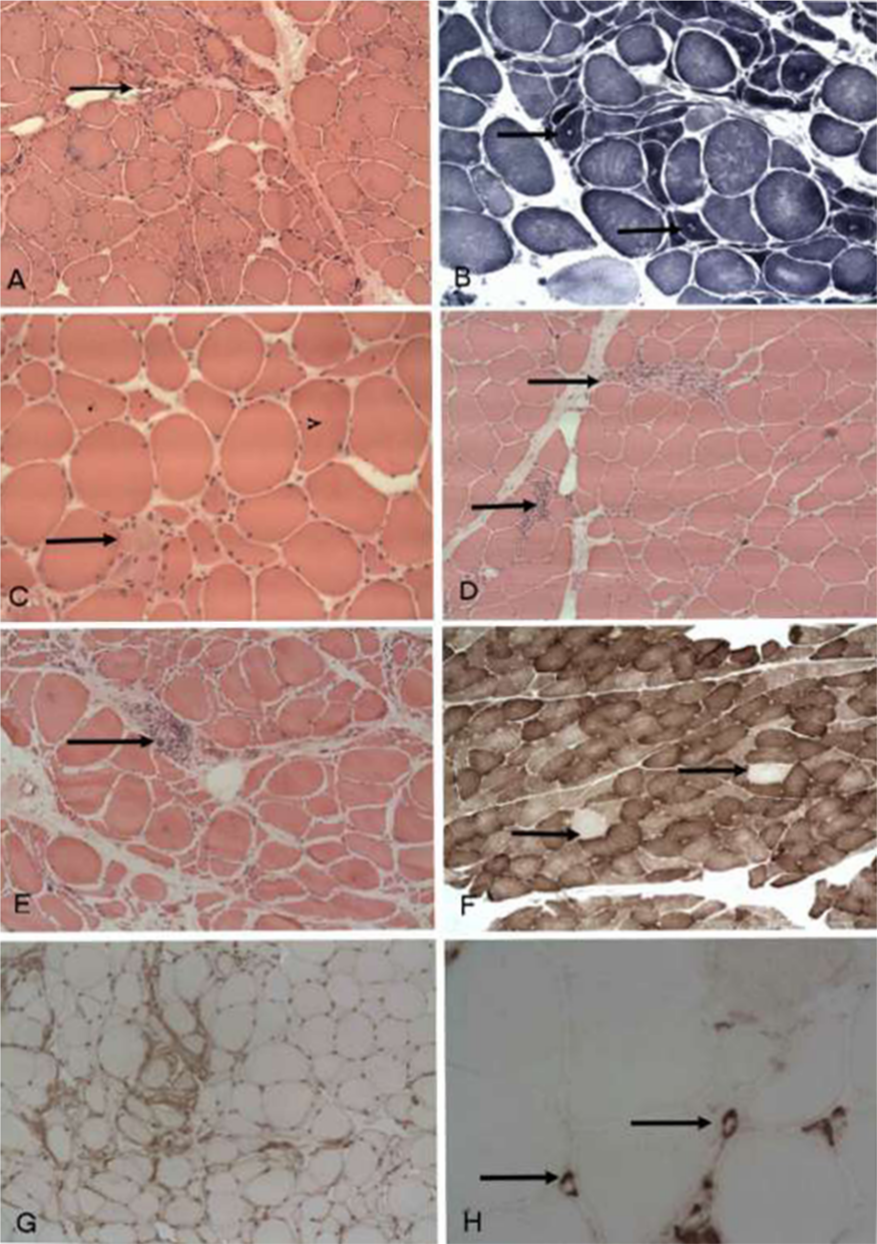
**Pathological changes in the muscles from ALS patients. A**: Low power view of muscle with neurogenic changes showing small and large group atrophy (arrow). **B**: NADH-TR stain showing atrophic and hyperdense fibres and target fibres (arrow). **C**: Fibre necrosis (arrow). **D**: Inflammatory cell infiltration in the endomysium (arrow) and **E**: in the perimysium (arrow). **F**: Cytochrome oxidase negative fibres (arrow). **G**: Patchy over expression of HLA) - ABC in muscle fibres. **H**: Deposition of compliment (C5-9) in the endomysial capillaries.

The extent of neurogenic atrophy differed between patients: small angulated fibres were observed in 29/31 (93.5%) cases, small grouped atrophy in 22/31 (71%) cases, (Figure 1A), pyknotic nuclear clumps in 13/31 (41.9%), increased internalized nuclei in 8/31 (25.8%) cases and fibre type grouping in 18/31 (58%) cases. There were NADH densely stained fibres in 27/31 (87%) cases; targetoid fibres in 20/31 (64.5%) cases (Figure 1B); and moth-eaten fibres in 13/31 (41.9%) cases.

### Muscle fibre necrosis and COX negative fibres

Muscle fibre necrosis was seen in five biopsies (16%) (Figure 1C); only one patient showed increased in CK level to 1386, two patients with inflammatory infiltrate and three with uniform over expression of HLA-ABC. The chronic mononuclear inflammatory cell infiltration was observed in five (16%) patients biopsies (Figure 1D and E), two of which also showed fibre necrosis, two with HLA-ABC over expression and two patient with increased CK level to 936 and 1386. In four biopsies there was more than 1% increase in the proportion of COX negative fibres (Figure 1F) with rates of 2- 3% . These cases also contain 1–3 ragged red fibres in the entire biopsy of each case. Although ragged red fibres are useful in showing abnormal accumulation of mitochondria, the percentage of COX negative/SDH positive fibres was more sensitive in identifying fibres with mitochondrial abnormality in muscle biopsy.

### Other changes

Cytoplasmic bodies presented in the form of globular, irregular, homogenous bodies, best demonstrated on the Gomori trichrome stain were present in 13/31 (41.9%) cases.

### Immunohistochemistry

The inflammatory infiltrate is mainly composed of lymphocytes and few macrophages. The lymphocytes are predominantly CD3 positive (T lymphocytes). The amount of inflammatory infiltrate was small in the deeper sections for the immunostainings for proper quantitative assessment but there is slight increase of CD4 over CD8 positive T lymphocytes Immunohistochemistry showed uniform and sarcolemmal over-expression of HLA-ABC (Figure 1G) in 5/31 (16%) but only few scattered small fibres positive with neonatal neomysin; therefore excluding HLA-ABC over expression was due to fibre regeneration and degeneration. Those five muscles with HLA over expression include two cases necrosis and one case with a predominantly chronic mononuclear inflammatory cell infiltration.

In 21/31 (67.7%) muscle biopsies there was granular deposition of the compliment (C5b-9) in the sarcolemma of the atrophic fibres consistent with fibre degeneration. However there were 3/31 cases where the C5b-9 antibody deposition was intense in the endomysial capillaries (Figure 1H) in addition to the presence of an inflammatory cell infiltration suggesting activation of compliments.

In the immunohistochemical stains, p62 was positive in 8/31 (25.8%) cases, displayed as faint, irregular and granular deposits at the sites of cytoplasmic bodies. None of the cases show abnormal TDP-43 immunoreactivity or over expression of lysosomal enzyme such as acid phosphatase; therefore the appearances of p62 reflected a non-specific deposition in fibre degeneration due to neurogenic changes.

Plasma samples were collected at the time of biopsy, but assessed for CK levels only after prolonged storage of the samples (>1 year), so results have an indicative value rather than providing an absolute quantification.

In three patients there was a marked increase in the serum CK level (807 IU/L, 1386 IU/L and 963 IU/L). In two of these cases, the muscle biopsy contained inflammatory cells and myofibre necrosis. The CK was mildly increased (200–500 IU/L) in 12/31 patients; in one of these patients an inflammatory cell infiltrate was present. The remaining patients had normal serum CK levels.

The Mann Whitney test shows no significant association between CK levels and inflammation (P = 0.33) and muscle fibre necrosis (P = 0.35).

In the controls all 20 of the muscle biopsies appeared normal. There was no evidence of neurogenic atrophy, myofiber necrosis or inflammation. The immunohistochemical staining for HLA Class I and C5b-9 was also negative in all fibres. In 3/20 biopsies there were occasional COX negative fibres amounting to less than 1% of total fibres, within normal limits attributed to age-related changes. Immunohistochemical stains for p62 and TDP-43 were negative.

## Discussion

In this large collection of muscle biopsies from patients with ALS, the predominantn pathological finding is neurogenic atrophy with a spectrum of findings due to denervation and re-innervation [34]. The most common feature of such neurogenic changes is small angulated and NADH-TR hyper-dense fibres. Additional features are fibre type grouping in nearly 50% of the biopsies and target fibres in 60% of the biopsies. In many muscle biopsies there is also evidence of disruption of intermyofibrillary architecture. Fibre type grouping resulting from re-innervations of fibres is only present in approximately 50% of our cases. On the other hand the NADH-TR stain appears to be useful in establishing the neurogenic changes in showing hyper-dense fibres and targetoid appearances which is present in 60% of the cases (Figure 1B). Other findings are an increased number of fibres with internalized nuclei and pyknotic nuclear clumps. The ATPase stains confirmed the atrophic fibres are of both types and not selectively type 2. Though it has been reported that type 2 fibres are affected by denervation more rapidly than type 1 fibres in early stages of the disease process [34], the lack of selective involvement of fibre type 2 in our cohort may reflect more chronic disease.

In addition to acute and chronic neurogenic atrophy, a few cases in our cohort demonstrated additional pathology less typical of denervation, such as fibre necrosis, low grade inflammation, and mitochondrial abnormalities. Albeit infrequent, these changes were not seen in our cohort of age-matched control biopsies. They are, nonetheless interesting as alternative theories are raised regarding the pathogenesis of ALS and involve the peripheral rather than the central nervous system. The neuronal degeneration in the upper and lower motor neurons in the CNS in ALS may not be the only abnormality and other cell abnormalities, such as in the muscles or changes at the neuromuscular junction may play a pathogenic role [1,22,35-39].

Four of our cases show evidence of mitochondrial abnormalities with an increased proportion of cytochrome oxidase negative fibres (Figure 1F) and few ragged red fibres beyond that seen in the age-match controls. Mitochondrial alteration has been reported in association with aging [27,40]; however in these four patients, the number of CXO negative fibres is seen in higher proportion (2-3%) expected in aging process and three of patients were in their 60s. This finding is in keeping with other reports of mitochondrial abnormality in the muscle of sporadic ALS patients and do not correlate with aging [15,17,23,41,42]. Mitochondria are known to be involved in the cellular production of oxygen radicals which play an important role in cell damage via oxygen/free radicals in many neurodegenerative diseases [1,16,43-45]. There is some evidence of involvement of mitochondria in the pathogenesis of the ALS. For instance, in experimental SOD1 mutant mice, there are reports of abnormal aggregates of mitochondria with vacuolation in skeletal muscle as well as in the anterior horn cells [18] and dysfunction of mitochondria in muscle, spinal cord and liver. Mitochondrial function has an important role to play in the pathogenesis of the some of the familial ALS like SOD1 mutation which appears to have toxic gene of function mutation in copper zinc super-oxide dysmutase, cytcolic and mitochondrial protein [36,43,46]. However, the nature of any contribution of mitochondrial abnormalities to human ALS remains uncertain. Abnormal mitochondrial functions have been reported in lymphocytes in ALS [11] and mitochondrial DNA deletions in ALS patients have been described [29,42]. There are also case reports of ALS and mitochondrial myopathy [12,16,32,41]. However Echaniz-laguna et al., [23] and Grehl et al. [47] reported that normal mitochondrial function is progressively altered with the disease’s progression secondarily. Recently, Bradley et al. [37] found no difference in respiratory chain enzyme and deleted mitochondrial DNA between ALS and controls. The finding of mitochondrial abnormalities in the biopsies of few of the ALS patients in this cohort and other published studies suggest some link whether primary or secondary to the pathogenesis of ALS or the ultimate motor neuron injury [44,48]. It is possible that mitochondrial abnormality in the skeletal muscle of sub-group of ALS patients which does not correlate with the age and may support the hypothesis that at least in such cases, mitochondrial dysfunction plays a role in the pathogenesis of the disease. Subclinical mitochondrial dysfunction in subgroups of ALS could also be present which is otherwise undetectable in the resting condition and become manifested with further external and environmental stress such as inflammation and may contribute to defective axonal transport at neuro muscular level and to retrograde neuronal degeneration in few patients.. This suggestion is supported by evidence from Siniliano et al. who reported increased lactate levels in patient with ALS after exercise [45].

Another interesting finding in muscle biopsies from our cohort of ALS cases is the presence of fibre necrosis and low grade inflammatory changes (Figure 1C, D and E). In five cases, there was evidence of muscle fibre necrosis; a rare but reported finding in neurogenic atrophy. Interestingly two of these cases showed an inflammatory cell infiltration and relatively high serum CK level. However we did not find association between presence of mild inflammation, increased CK levels and clinical manifestation and progress of the patient. The inflammatory process, including the up regulation of HLA- ABC and compliment deposition (C5b-9) could also be secondary to neurogenic changes and secondary myofiber necrosis. These cases show no evidence for endomysial inflammation with invasion of non-necrotic fibres as seen in inflammatory myopathies or inclusion body myositis IBM. However, a finding of low grade inflammation in the muscle is intriguing given evidence that inflammation in the central nervous system may be a contributory factor in ALS pathology. There is mounting evidence that activated microglia and astrocytes, endothelium and lymphocytes trafficking in the CNS of ALS patients may play an etiologic role through the production of molecules, like IL-6 and TNFalpha and increased oxidative stress [49-52]. Our results may not indicate a primary muscle disease or inflammation, but it support the view that the low grade inflammation and sub clinical mitochondrial alteration in the muscle of some ALS patients may add further burden in damaging the neuromuscular junctions and contribute to “dying back” of motor neutrons. The interaction between the peripheral and central inflammatory process is critical to understand the its pathogenesis It has been theorized that ALS pathology may not necessarily be directly due to motor neuron degeneration but may start more distally at the neuromuscular junction. Fischer et al. [53] showed that the alteration of the neuromuscular junction led to axonal degeneration and ultimately motor neuron degeneration in the CNS. Evidence showed that a reduction in NOGO-A, a neurite outgrowth inhibitor led to increase in the lifespan of SOD1 mice [54,55]. In addition Bradley et al. [37] showed that myoblasts from ALS patients exhibit an enhanced sensitivity to oxidative stress. This evidence suggests that ALS muscle may be particularly vulnerable to additional stress and activate an apoptotic process [37,56]. Such oxidative stress may come through different routes such as environmental or even inflammatory myopathy [39,49,57]. It is possible that in some ALS patients’ alterations in muscle and the neuromuscular junction may be pathogenic contributors resulting in retrograde degeneration of the motor neurons. ALS is a complex and multifactorial disorder with many potential etiologic and pathogenic mechanisms resulting in dysfunction at the neuromuscular, axonal and neuronal levels [1].

Finally, many muscles in this series showed a cytoplasmic body which is a known alteration in the intermyofibrillary article true in long standing neurogenic changes expected in ALS patients. Also few cases showed p62 granular deposits but with no over expression lysosomal enzyme such as acid phosphatase. The appearances are not specific and reflect process of degeneration in the muscle fibres. There is no specific well define inclusions and all the sections were negative with TDP43 stain.

## Conclusion

In conclusion, acute or chronic neurogenic atrophy is the nearly uniform finding in this large cohort of muscle biopsies of ALS patients. In addition, low grade endomysial and perimysial chronic inflammation and mitochondrial alterations are seen in a small proportion of muscle biopsies from patients with sporadic ALS beyond that seen in age-matched control biopsies. These findings do not exclude the diagnosis of ALS. Neither do these findings meet criteria for primary inflammatory muscle disease or mitochondrial cytopathy. However, these findings could reflect contributing pathogenic mechanisms whereby additional oxidative stress by inflammation or other factors could unmask subclinical mitochondrial dysfunction in the muscle of some ALS patients. These muscle biopsy findings from a relatively large ALS patient cohort, could provide additional clues to understanding the seemingly multifactorial, complex pathogenesis of ALS.
